# Case Report: Systemic reactive angioendotheliomatosis in a young cat with a history of esophageal strictures and balloon dilations

**DOI:** 10.3389/fvets.2026.1778392

**Published:** 2026-04-14

**Authors:** Tara Hammond, Christine Savidge, Gisela Martinez-Romero

**Affiliations:** 1 Department of Emergency and Critical Care, Tufts Veterinary Emergency Treatment & Specialties, Walpole, MA, United States; 2 Department of Internal Medicine, Tufts Veterinary Emergency Treatment & Specialties, Walpole, MA, United States; 3 Department of Comparative Pathobiology, Cummings School of Veterinary Medicine at Tufts University, North Grafton, MA, United States

**Keywords:** anemia, esophageal stricture, feline, feline systemic reactive angioendotheliomatosis, seizures, thrombocytopenia

## Abstract

A 3.3-year-old castrated male domestic shorthair cat presented to the emergency service with neurologic signs. The cat had an extensive medical history, including previous treatment for esophageal strictures via balloon dilation. Diagnostics revealed a coagulopathy, a highly regenerative anemia, and marked thrombocytopenia. The cat’s neurologic signs progressed rapidly, culminating in seizures and cardiac arrest. A necropsy revealed findings consistent with feline systemic reactive angioendotheliomatosis (FSRA), a rare, idiopathic, multisystemic, and uniformly fatal disease. To date, only 16 cases of FSRA have been reported in the veterinary literature, and very few provide detailed descriptions of attempted treatments. It is theorized that repeated tissue trauma from procedures, infection, and chronic inflammation may have triggered a more systemic response, contributing to the development of FSRA in this cat. Further investigation is needed to better understand this rare disease.

## 1 Introduction

Feline systemic reactive angioendotheliomatosis (FSRA) is a rare, idiopathic disease that causes multisystemic disease in cats. The disorder is characterized by intravascular proliferation of spindle cells, leading to thrombosis, hemorrhage, and necrosis in the affected organs. The heart, brain, and lungs are most often affected, although several other organs, including the kidneys, liver, spleen, gastrointestinal tract, urinary bladder, eyes, and lymph nodes, can also be affected (1–7). Once systemic signs develop, the disease progresses rapidly and is uniformly fatal.

To date, only 16 cases of FSRA have been reported in the veterinary literature (1–7). Affected cats most commonly present with dyspnea, although some develop neurological signs. Historical signs such as lethargy, anorexia, weight loss, or behavior changes may be vague. Reported cases involve cats aged 9 months to 14 years old, with a median age of 3.5 years. Multiple breeds have been affected, and 75% of reported cases have been male (1–7). Cats with FSRA often present with anemia, thrombocytopenia, hyperbilirubinemia, mild alanine aminotransferase (ALT), and aspartate aminotransferase (AST) elevations, and marked increases in creatine kinase (CK). The heart is the most commonly and severely affected organ on necropsy, with the greatest changes observed in the small arterioles of the myocardium (1–7). Among reported cases, a full history, clinical signs, results of diagnostics, and attempted treatments are described in detail in only two cases (6, 7). This likely reflects that the disease is typically advanced by the time clinical signs present, as affected cats often die within hours to days of onset. The duration of the disease process before outward clinical signs are manifested remains unclear. There are currently no early known markers for FSRA. Identification of such markers could enable earlier diagnosis and may potentially improve treatment strategies and outcomes in the future. The purpose of this case report is to provide a detailed account of the clinical progression, comprehensive diagnostic findings, attempted treatments, and necropsy results in this rare case of FSRA in a young cat previously treated for esophageal strictures.

## 2 Case description

A 3.3-year-old castrated male domestic shorthair cat presented to the emergency service at Tufts Veterinary Emergency Treatment & Specialties after being found laterally recumbent in a pool of urine. The owner reported that the cat was vocalizing and appeared unaware of his surroundings. He had also been lethargic and anorexic for the preceding 48 h. The cat had been living on a boat and traveling along the East Coast of the United States and throughout the Caribbean.

**Figure 1:**
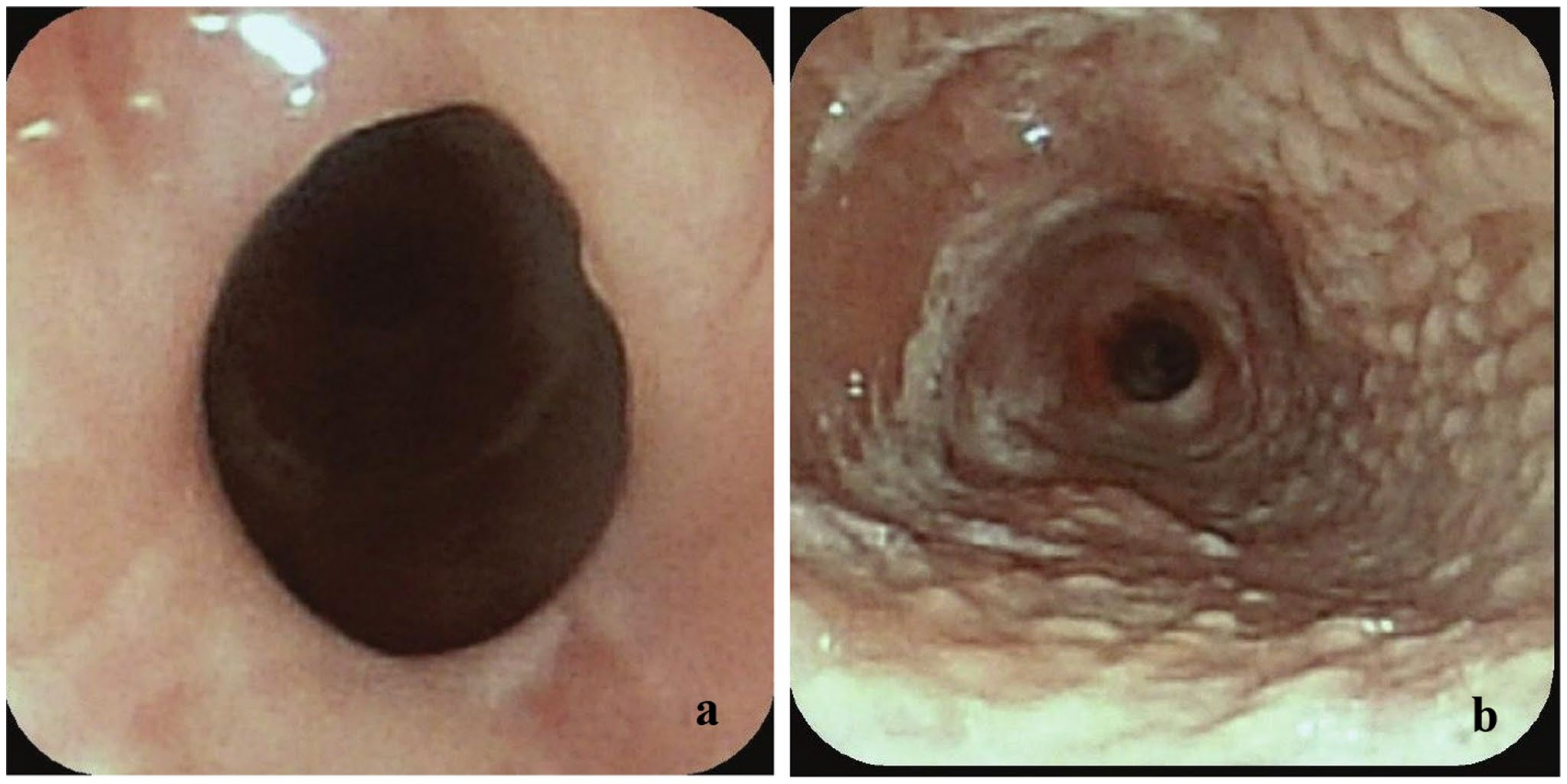
**(a)** Esophagoscopy image identifying a 7–8 mm diameter stricture 15 cm aborad (near heart base). Size estimated as endoscope could not pass through the stricture without trauma. **(b)** Esophagoscopy image with endoscope position at 18 cm aborad identifying severe esophagitis and a 3–4 mm stricture visible at 20 cm aborad.

**Figure 2:**
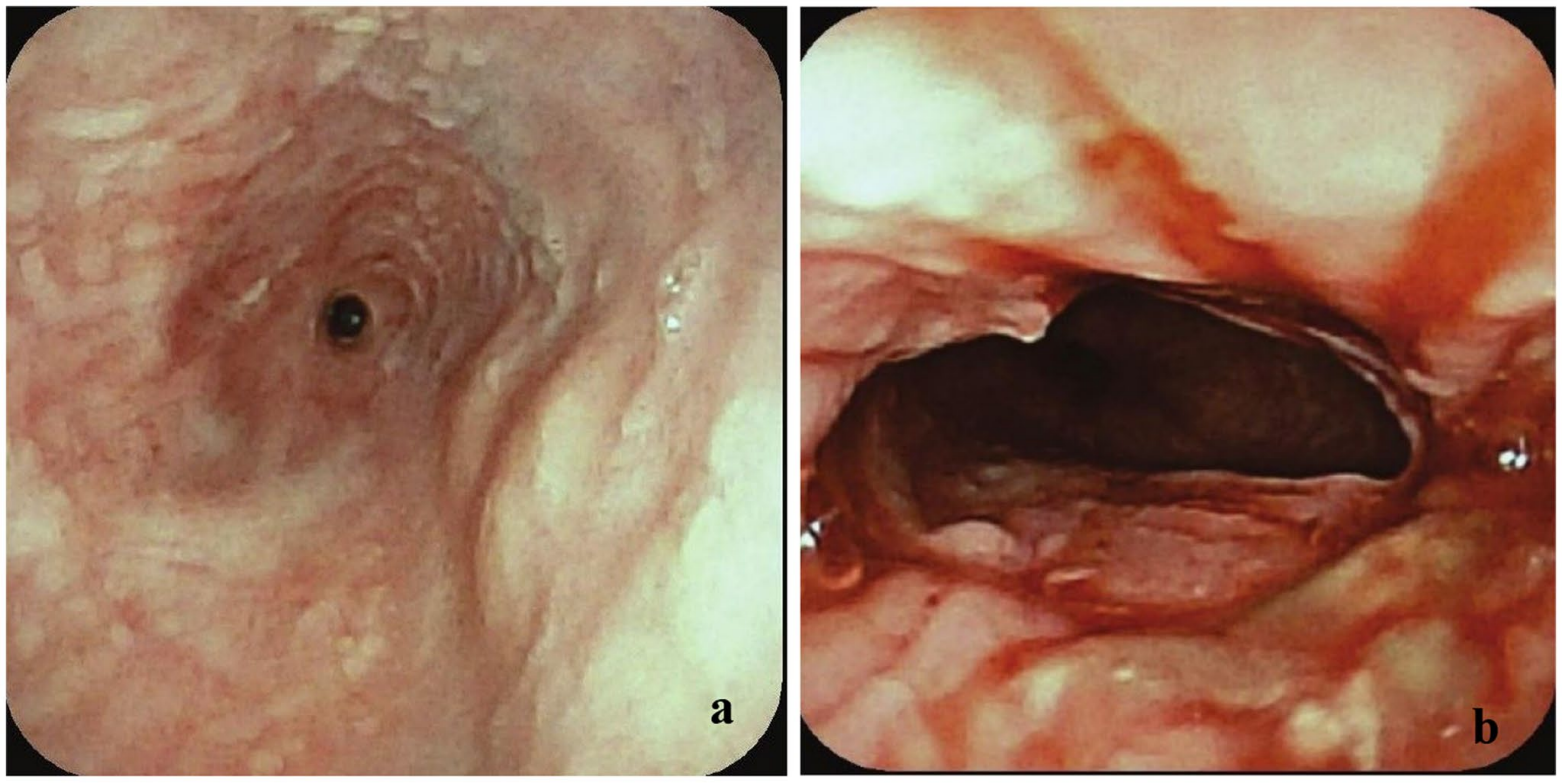
**(a)** Esophagoscopy image with endoscope position at 16 cm aborad identifying a 3–4 mm stricture at 20 cm aborad with improved esophagitis. **(b)** Esophagoscopy image post-balloon dilation of the stricture at 20 cm aborad after dilation to 9 mm. Moderate bleeding and tissue trauma were observed.

The cat had an extensive medical history. Two and a half years prior, he had been brought to a rescue organization in an emaciated state with frequent regurgitation and episodes of dyspnea after eating. Thoracic computed tomography (CT) identified a sliding hiatal hernia with no evidence of a vascular ring anomaly. Esophagoscopy using a small flexible video gastroscope (Fujifilm EG-530N2, 5–9 mm) revealed severe esophagitis and secondary esophageal strictures located approximately 15 and 20 cm aborad (Figures 1a,b). Initially, the rescue elected medical management with omeprazole (1 mg/kg PO q12, compounded 10 mg/mL Mixlab New York, NY), sucralfate (80 mg/kg PO q8, Amneal Pharmaceuticals LLC, Bridgewater, NJ, United States), and slurry feedings; however, clinical signs persisted. The cat was subsequently adopted and underwent a left-sided incisional gastropexy a few weeks later. Postoperatively, repeat esophagoscopy showed improvement in esophagitis (Figure 2a), and esophageal balloon dilation was performed, dilating the proximal stricture to 10 mm and the distal stricture to 9 mm (Figure 2b). The procedure was repeated 1 week later, achieving dilation of the proximal stricture to 13.5 mm and the distal stricture to 12 mm. Moderate bleeding and tissue trauma occurred with each procedure, but there was no evidence of perforation.

**Figure 3:**
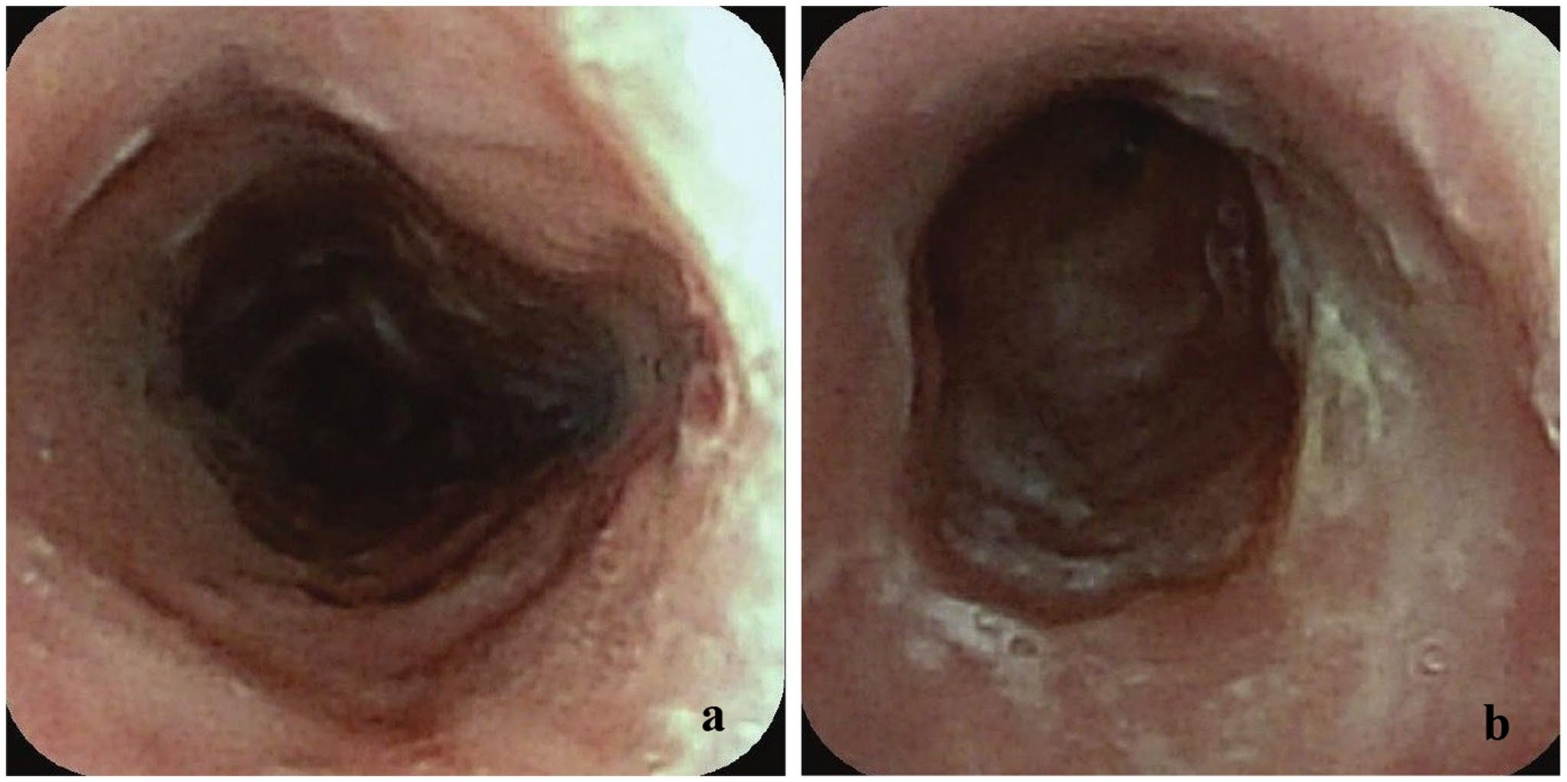
**(a)** Esophagoscopy image after B-tube dilation to 15 mm and removal with endoscope position at 14 cm aborad. **(b)** Esophagoscopy image after B-tube dilation to 15 mm and removal with endoscope position at 19 cm aborad.

Following each dilation procedure, the cat was maintained on omeprazole and carafate with topical prednisolone (0.16 mg/kg PO q24; compounded, Mixlab, New York, NY, United States) as well as a 3-day course of buprenorphine (0.02 mg/kg SL q8; Buprenex, Epicur, Mount Laurel, NJ, United States). Six weeks later, the patient presented again with persistent regurgitation, lethargy, anorexia, and fever (103.6 °F). Chest radiographs revealed a mild diffuse interstitial pattern; early aspiration pneumonia could not be ruled out. Given the cat’s high risk, he was treated with amoxicillin and clavulanate potassium (13.75 mg/kg PO q12, Clavamox, Zoetis Inc., Kalamazoo, MI). Repeat esophagoscopy was performed, which identified a 4-mm stricture approximately 20 cm aborad; the more proximal esophagus appeared normal. The stricture was again dilated using a series of balloon dilators from 4 to 13.5 mm, resulting in moderate but acceptable hemorrhage. After the procedure, a 14-Fr, 15-cm-long, 15-mm dilation-sized esophageal balloon-dilation feeding tube (B-Tube, MILA International, LLC, Florence, KY, United States) was placed as previously described in the veterinary literature (8). Correct placement was confirmed via fluoroscopy. The cat remained hospitalized for twice-daily balloon dilations and esophageal tube feedings for 8 days after placement, after which the owner took over ongoing treatments, including twice-daily dilations. The cat made good progress but developed a tube-site infection 5 weeks after placement, which cultured a resistant *Pseudomonas aeruginosa* and *Klebsiella* spp. The cat was treated with enrofloxacin (5 mg/kg PO q24, Baytril, Elanco Animal Health, Indianapolis, IN, United States) for 14 days. The tube was removed 6 weeks after placement. Repeat esophagoscopy at the time of removal showed resolution of the strictures and esophagitis (Figures 3a,b). The owner reported that the cat made a full clinical recovery and was reported to be doing well on rechecks.

Twenty-three months later, the cat experienced the acute neurological event noted above and was immediately brought to the emergency room. On physical examination in the emergency room, the cat was quiet but alert. He had a heart rate of 180 beats/min, a respiratory rate of 60 breaths/min, and a temperature of 100.5 °F. His gums were pale. He was at an optimal body condition score and weighed 4.9 kg. The cat was ambulatory and alert, with no cranial nerve deficits.

## 3 Diagnostic assessment, therapeutic intervention, and outcome

A complete blood count (CBC) revealed a highly regenerative anemia with a hematocrit (Hct) of 24.9% (normal, 30.3–52.3%), an absolute reticulocyte count of 341.8 K/μL (normal, 3.0–50.0 K/μL), and a severe thrombocytopenia of 5 K/μL (normal, 151–600 K/μL). A chemistry profile revealed a mild hypokalemia of 3.3 mmol/L (normal, 3.7–5.9 mmol/L) and an ALT of 118 U/L (normal, 12–115 U/L). No spherocytes, autoagglutination, or red blood cell parasites were identified on the blood smear; occasional reactive lymphocytes were observed. A feline leukemia virus/feline immunodeficiency virus enzyme-linked immunosorbent assay (FeLV/FIV ELISA) test was negative. Chest and abdominal radiographs were unremarkable, as was a thoracic and abdominal point-of-care ultrasound. The cat was admitted to the hospital and started on lactated Ringer’s solution (LRS, Baxter Laboratories, Deerfield, IL, United States) with 20 mEq KCl/L (potassium chloride, Hospira, Inc., Lake Forest, IL, United States) at 60 mL/kg/day, along with maropitant (1 mg/kg IV q24; Cerenia, Zoetis Inc., Kalamazoo, MI, United States).

The next morning, the cat was quiet but alert and responsive with stable vital signs. Systolic blood pressure was 192 mmHg. Approximately 1 h later, he became stuporous and tetraparetic and began circling to the right. He was non-visual and had some isolated facial twitching. He lost the menace reflex in the left eye. The entire event lasted approximately 60 s, and he remained in a seemingly post-ictal state for approximately 5 min before returning to normal. During the episode, his heart rate was 180 bpm, and systolic blood pressure, measured immediately afterward, was 204 mmHg. The cat was suspected to have had either multifocal or diffuse intracranial disease, and it was likely that the owner had found him in a post-ictal state the day before.

Levetiracetam (20 mg/kg IV q8; Keppra, Eugia US LLC, E. Windsor, NJ, United States) was initiated. Additionally, doxycycline (10 mg/kg IV q24; Fresenius Kabi, Lake Zurich, IL, United States) and clindamycin (10 mg/kg IV q12; Pfizer Inc., New York, NY, United States) were administered pending the results of infectious disease testing, given the cat’s extensive travel history. Comprehensive whole blood polymerase chain reaction (PCR) testing for infectious diseases, including feline calicivirus, *Cytauxzoon felis*, *Bartonella* spp., *Anaplasma* spp., *Ehrlichia* spp., feline coronavirus, *Mycoplasma haemofelis*, *Candidatus Mycoplasma haemominutum*, *Candidatus Mycoplasma turicensis*, FeLV, FIV, *Cryptococcus* spp., *Salmonella* spp., *Toxoplasma gondii*, and feline panleukopenia, later yielded negative results.

During the second day of hospitalization, the cat experienced two additional neurological episodes, during which his pupils became fixed and mydriatic. He appeared non-visual, drooled, lifted his right paw in a stiff extension, urinated, and exhibited mild focal facial twitching. He was unresponsive to noxious stimuli during the events. Each episode lasted approximately 1–2 min, and he returned to normal within 5–10 min.

The cat was considered too unstable for anesthesia and at a high risk of hemorrhage to proceed with magnetic resonance imaging (MRI) or CSF tap, respectively. Given these limitations, the owner opted for ongoing critical care, and dexamethasone sodium phosphate (0.25 mg/kg IV q24; MWI, Boise, ID, United States) was started in the event his disease was immune-mediated. The levetiracetam dose was also increased (30 mg/kg IV q8). After a third seizure, the cat developed ecchymosis on his ventral abdomen and began to have hematuria. Shortly thereafter, he vomited blood-tinged fluid. Repeat laboratory evaluation showed a worsening anemia (Hct 20%), ongoing thrombocytopenia (13 K), leukopenia [4.0 K (normal 4.5–15.7)] with borderline neutropenia [2.2 K (normal, 2.1–10.1 K)], and normal blood glucose, lactate, and electrolytes. The ammonia level was 0 μmol/L (normal, 0–95 μmol/L). A Coombs test later came back negative. Prothrombin time (PT) and partial thromboplastin time (PTT) showed mild to moderate prolongations at 24.0 s (normal, 13.0–22.0 s) and 154 s (normal, 65–119.0 s), respectively. Pantoprazole (1 mg/kg IV q12; Protonix, Piramal Critical Care, Inc., Bethlehem, PA) and phytonadione (2.5 mg/kg PO q12; Vitamin K_1_, MWI, Boise, ID) were initiated. The cat was cross-matched and started on approximately 7.5 mL/kg of compatible type A packed red blood cells. Post-transfusion, the packed cell volume (PCV) was 27%, and the cat appeared brighter and neurologically normal.

The next day, the cat had three more episodes similar to the previously described neurological events. Repeat point-of-care laboratory work showed stable clotting times, PCV/TS, and blood glucose. His serum appeared icteric. The cat then had two additional neurological events and became obtunded. He was concurrently bradycardic (140 bpm) and hypertensive (systolic blood pressure 200 mmHg), raising concern for elevated intracranial pressure and impending brain herniation or death. Given concerns of imminent arrest, mannitol was administered (1 g/kg IV; ICU Medical Inc., Lake Forest, IL, United States). Within 15 min, the cat was much brighter and neurologically normal. A phenobarbital loading dose was planned once additional IV access could be attained, but venous access had become increasingly difficult due to thrombocytopenia, coagulopathy, and previous venipunctures. Unfortunately, the cat suffered acute cardiac arrest a few hours later. Cardiopulmonary resuscitation was started, but efforts were ultimately terminated per the owner’s wishes due to the poor prognosis.

The cat was sent for postmortem examination. Gross examination revealed generalized icterus, pulmonary edema, and hemorrhages of the urinary bladder mucosa. The mucosa of the small intestine had extensive dark red areas, and the lumen contained hemorrhagic fluid. The distal esophagus was segmentally dilated with a stricture. The heart weighed 24 g. The left ventricular wall had concentric thickening, and the posterior interventricular septum and the adjacent posterior left ventricle contained a transmural area of hemorrhage.

**Figure 4:**
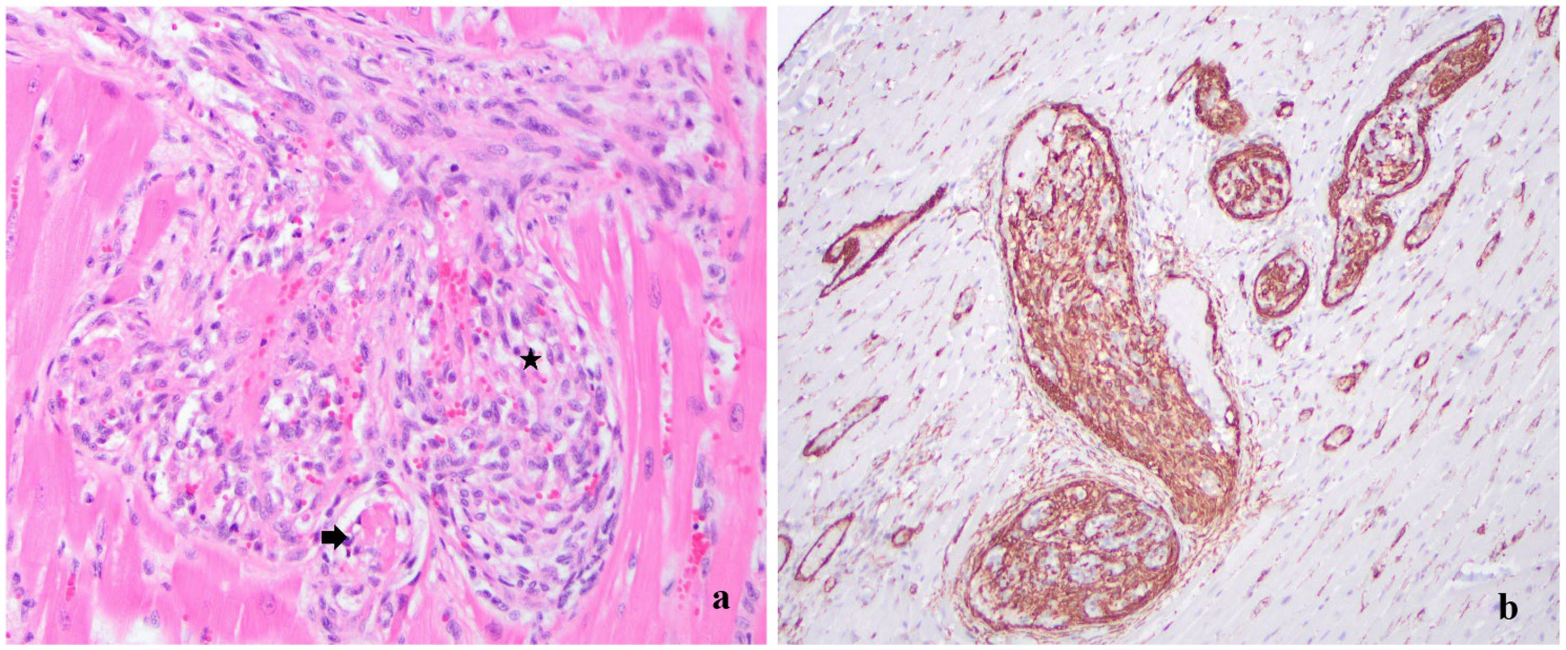
**(a)** Heart, left ventricle. Hematoxylin and eosin stain, 20×. The lumen of the arterioles is occluded with spindle cells concentrically arranged (asterisk). Within the spindle cell proliferation, there are small channels filled with erythrocytes or fibrin thrombi (arrow). **(b)** Heart, left ventricle. Immunohistochemistry, 10×. Intraluminal spindle cells forming glomeruloid intravascular structures display strong cytoplasmic immunopositivity for smooth muscle actin.

Histologically, the lumens of numerous arterioles in the ventricular myocardium, interventricular septum, brain, kidney, small intestine, esophagus, pancreas, lymph nodes, spleen, and lungs were completely occluded by spindle cells concentrically arranged, forming glomeruloid structures. The spindle cells had scant eosinophilic cytoplasm, oval-to-elongated nuclei with finely stippled chromatin, and a single nucleolus. Several affected arterioles contained fibrin thrombi and were surrounded by areas of hemorrhage (Figure 4a). No vasculitis or necrosis of the vascular wall associated with fibrin thrombi was observed.

Multiple patchy areas of fibrosis were found in the myocardium near the intravascular spindle cell proliferations. The proliferating spindle cells in the myocardium displayed strong cytoplasmic immunoreactivity for smooth muscle actin (SMA) and Factor VIII (Figure 4b).

In the esophagus, the submucosa contained mild to moderate lymphocytes and fibrosis, which separated and replaced smooth muscle cells. These regions corresponded to the grossly described esophageal strictures. Additionally, there were splenic extramedullary hematopoiesis, bone marrow myeloid, erythroid, and megakaryocyte hyperplasia, and mild cholestasis in the liver. The microscopic and immunohistochemical findings support a diagnosis of FSRA.

## 4 Discussion

Angioendotheliomatosis is the proliferation of cells within the vascular lumen, leading to thrombi and occlusion. This proliferation can be due to a malignant or benign (reactive) process. In malignant angioendotheliomatosis (currently termed angiotropic or intravascular lymphoma), neoplastic lymphocytes proliferate within vascular lumens and can affect almost all major organs. It is rapidly fatal in humans and animals (9–12). Seventeen cases of intravascular lymphoma have been described in dogs, and one case in a cat (11, 13). This differs from reactive angioendotheliomatosis, as observed in this case, where non-neoplastic spindle cells proliferate and occlude vascular lumens in glomeruloid patterns with fibrin thrombi deposition.

Given the severe, rapidly progressive nature of FSRA, it is usually impossible to obtain an antemortem, definitive diagnosis in these cases. Even if a histological diagnosis was obtained antemortem, there are no guidelines to follow since no effective treatment is known. Even if a histological diagnosis were obtained antemortem. Clinicians are challenged to manage clinical signs and to treat for other diseases on the differential list in real time. Given that FSRA most profoundly affects the heart, brain, and lungs, among other organs, many cats have been misdiagnosed and/or received therapies targeted at immune-mediated disease, infectious disease, hypertrophic cardiomyopathy (HCM), and/or meningoencephalitis before death, as in our case. There are only two case reports in the veterinary literature describing the clinical progression of a cat ultimately diagnosed with FSRA, while the remaining reports focus solely on postmortem findings (6, 7). The first, a 10-year-old male cat, presented with vomiting, lethargy, anorexia, tetraparesis, cranial nerve deficits, seizures, and dyspnea, and was suspected of having primary neurological disease (7). The cat underwent a full workup but failed to respond to treatment and was euthanized 6 days into treatment. The other case report describes a 2-year-old castrated male cat with neurological signs, initially suspected to have HCM based on echocardiography. The cat was euthanized 2 days into treatment due to clinical deterioration and was found to have FSRA on necropsy (6).

The cat in this case report was young and male, which is in line with previously reported cases, but he presented primarily with neurologic signs, which are less common. The vast majority of cats present with dyspneic or congestive heart failure. This cat also exhibited clinicopathologic changes associated with FSRA, including a Coombs-negative, regenerative anemia, coagulopathy, and thrombocytopenia, as well as a mildly increased ALT and, later, total bilirubin. Similar to most other cases, the cat died due to disease progression within days of presentation. The diagnostics and attempted treatment, although futile, are discussed in detail here in an effort to share greater insight into the clinical course of this devastating disease.

Since knowledge about FSRA is limited, it is theorized that the repeated anesthetic events and tissue trauma with surgery, balloon dilations, and feeding tube placement, combined with infection and chronic inflammation, may have induced a systemic reactive response in this cat, leading to the FSRA. The cat in this case report had classic histopathologic lesions associated with FSRA, including proliferation of spindle cells in a glomeruloid pattern that obstructed innumerable arteriolar lumens in several organs with thrombi formation (14). Furthermore, immunohistochemical staining in this case showed strong immunoreactivity for SMA and Factor VIII, which is in line with previous reports of FSRA (2, 5). This finding further supports the important distinction that FSRA is a reactive rather than a neoplastic disorder. It is unclear how long the intravascular spindle cell proliferation has been occurring in the body before these cats present with end-stage clinical signs. The cat in this case had those pathognomonic intravascular spindle cell proliferative lesions found within the strictured segments of the esophagus, lending further support to a possible cause-and-effect relationship. Even if the treatment for his esophageal disease had been known to increase his risk of FSRA, treatment would not have been altered because the cat was profoundly affected by the regurgitation, esophagitis, and strictures, and FSRA is extremely rare.

Risk factors for the development of FSRA remain unclear. There is a lack of consistent risk factors related to infectious disease, and the role of genetics has not been studied. One study found that 4 of 4 cats with FSRA were PCR positive for *Bartonella henselae* and/or *Bartonella vinsonii*. *Bartonella henselae* infection and infection in cats have been associated with anemia, thrombocytopenia, eosinophilia, and hyperglobulinemia, as well as endomyocarditis, ocular disease, and neurological signs (15, 16). However, the cat in this case report was negative for *Bartonella* spp., as were the cats described by Yamamoto and Raven, so the relationship remains unclear.

As noted previously, it was not possible to obtain an antemortem diagnosis in this case. Emergent treatment was largely based on treating other, more common differentials and was made on a risk–benefit basis. The cat was treated for conditions that were later identified as not being present, such as immune-mediated disease and infectious disease. In critically reviewing this case, different choices might have been made if a definitive diagnosis had been available or if the diagnostic turnaround times were quicker. The cat was treated with mannitol when showing signs of elevated intracranial pressure and impending brain herniation or death. Given that mannitol can exacerbate coagulopathies and may, in theory, cause intracranial hemorrhage, if present, in retrospect, a trial of hypertonic saline might have been a preferable option. However, as noted previously, ongoing treatment proved futile with this inevitably fatal disease. It is also important to note that this cat was treated with a full course of enrofloxacin based on culture results. Although he had normal renal function and the dose prescribed was low, pradofloxacin may have been a safer alternative given the risk of retinopathy in cats treated with enrofloxacin.

In conclusion, FSRA remains a very rarely reported condition in cats. It is often only identified at necropsy. To the best of the author’s knowledge, there is no reported case that has had an antemortem diagnosis with successful treatment. Clinicians should be familiar with the disease and increase their clinical suspicion/testing/treatment. Further information is needed to better understand and manage this condition.

## Data Availability

The original contributions presented in the study are included in the article/supplementary material, further inquiries can be directed to the corresponding author.
